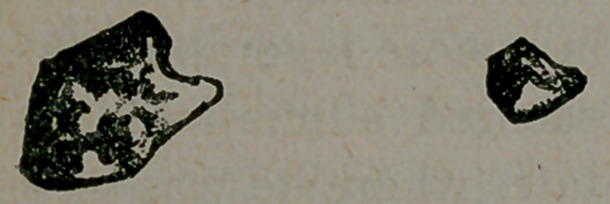# Carlsbad Water and the Sprudel Salt

**Published:** 1891-02

**Authors:** William F. Hutchinson

**Affiliations:** Providence, R. I.


### Selections.

### CARLSBAD WATER AND THE SPRUDEL SALT.

A CLINICAL STUDY, BY WILLIAM F. HUTCHINSON, M. D., PROVIDENCE, R. I.

In the north-west corner of Bohemia, some two thousand feet above the level of the sea, there lies a little hill town that climbs up sharply rising terraces on both sides of a swift mountain stream that is called the Tepl.

It nestles from passing sight in the heart of great forests of pine and beech trees, and is full of legends of knights and ladies fair, that have been visitors to its valley for six hundred years or more.

With but 12,000 of its own residents, it has a population of 30,000 every summer, the others coming from all parts of the world to avail themselves of its wonderful waters, that are so gifted with healing power.

These springs of Carlsbad are now so well and widely known throughout the civilized world and have attained such a high reputation everywhere that any extended description is not needed, and if it were, may readily be obtained from better pens than mine.

This paper is intended to call the attention of the profession to a few case records that I have collected from the mass that have accumulated during thirty years of constant use of these waters and their salts for a variety of diseases.

There is nothing more certain than that to obtain the best results from the therapeutic use of medical waters, they must be taken at the spot whence they emerge from the earth, where their administration can be controlled by the experience of resident physicians, and where Nature's forces of seclusion, rest and pure air are free to act as potent auxiliaries.

But for all the world, this is not possible; and, were the good that is done every year by the waters of these famous fountains alone, confined to sufferers who are fortunate enough to reach their home, great as that aggregate number is, only a small part of the large army of invalids that blesses their healing powers would ever have heard of them.

After comparing the effects of Carlsbad waters at the springs with those obtained from the same when taken at home in America, I am convinced that the only loss they sustain in removal, is that they do not bring in their neat bottles, the air and regime of the Bohemian mountain spa.

So much of the latter as concerns exercise, at least, may be followed anywhere; and I venture to say a few words about that.

Free movement is one of the principal conditions necessary, particularly for those whose sickness was originally chiefly caused by a sedentary life. Suitable muscular activity as directed by the family physician, promotes the excretion of decomposition products accumulated in the body by prolonged inactivity. Exercise, such as walking, out-door games, etc., must be moderate and selected for each case, and may readily be overdone. But when the patient's condition demands rest and confinement to bed, the waters are still of equal value, only they must be given in smaller doses.

Diet should be restricted while they are being taken, to non-nitrogenous foods, its daily quantity lessened, and the use of alcoholic stimulants prohibited. If digestion is disturbed, a little extract of malt may be administered with each meal. Diabitics, who are forbidden starches, will find an agreeable and effective substitue for wheat bread in the almond bread of Prof. Seegen, the formula for which is as follows:

Pound in a stone mortar, four ounces of blanched Jordan almonds to a smooth powder. Put this in a linen bag and boil for fifteen minutes. Mix thoroughly with three ounces of butter and two eggs, add the yolks of two more eggs with a little salt and beat well. The whites of three more eggs, beaten to a stiff foam, are next to be beaten into the dough and when formed into biscuits, they are to baked until well done in a slow oven.

Carlsbad waters are odorless, palatable and free from color, with a faint saline taste and never produce nausea, Even when taken in considerable quantities they produce no diarrhœa or feeling of discomfort. I once drank six tumblers within an hour without the slightest unpleasant effects.

They act directly upon the mucous membrane of the stomach and alimentary canal, and secondary as a powerful alterative; soothing irritated surfaces reached and changing blood from acid to alkaline re-action. During this process, all calculi of the former kind, whether biliary or cystic, are steadily dissolved, gouty concretions softened and placed in condition for absorption, and rheumatic deposits in muscles are removed. In diabetes mellitus, Carlsbad waters have long been considered as exercising a powerful curative influence, and I have personally known of cures made at the springs where other forms of treatment have failed.

Since the use of these waters and the Sprudel salt that goes with them is, in America, mainly confined to these two forms of disease, I shall cite only cases which are of one or the other class.

Case 1. A. B., physician, aged thirty-five years, of healthy parentage and a fine physique, had been systematically doing two men's work for several years in spite of all sort of protest from family and friends.

Some five years ago, present date 1890, he began to show signs of exhaustion and nervous tire, but still continued work of the most exposed character and fatiguing description, until the spring of 1886, when he was persuaded to take a trip to Europe for rest. While in England he visited Brighton, where he remained for several weeks taking the waters and baths; was there attacked by nephritic colic attended with excruciating pains of the severest description, accompanied with passage of several small stones.

Four months after his return Dr. B. consulted me, and I at once put him upon Carlsbad water as previously suggested, adding a prescription of my own which I have found to act well at a distance from the springs. It is hot Carlsbad baths daily at a temperature of 150° F. for ten minutes each time. These baths are made by adding eight ounces of Sprudel salt to an ordinary bath-tub of water, gradually increasing temperature until the desired heat is reached, and should be taken night and morning. Another and effective way of giving these baths is by means of vapor. The patient, nude, except for a loose blanket covering stool and person to the neck, is seated on a perforated stool, under which a shallow pan of Carlsbad previously saturated with Sprudel salt is slowly boiled away. Profuse perspiration follows and a rapid absorption of elements of the water as they are in turn volatilised, complete vaporisation being insured by combustion of the dry residue left after water has disappeared.

After a month of this treatment, Dr. B. passed a large number of calculi per urethram, and drawings were made of two of the largest which are here reproduced. All gouty symptoms disappeared at the same time; a recent clinical examination showed him free from uric acid urine and calculi, and his general health improved after the course.

Case 2. Mrs. S., of middle age and healthy family. For several years she had suffered with gouty rheumatism and slowly increasing concreations in finger joints, which were steadily growing and causing loss of motion. She was at last attacked by arthritic neuralgia, for which she consulted me and I found her system charged with uric acid.

I was unable to learn that any calculi had been voided, although all urine was loaded with brick dust and a copious deposit fell from a beaker full kept over night.

Mrs. S. was at once placed upon a course of Carlsbad water and the steam baths of Sprudel salt solution. Six tumblers of the water were ordered to be drank each day with a twenty minute bath morning and evening. Localized galvanism, descending nerve current, was administed for her neuralgia, which soon subsided.

After forty baths a distinct relaxation of solidity in the gouty concretions was noticed, and they began to disappear. The neuralgia was relieved after a week. In two months all joints were normal and clinical tests demonstrated absence of uric acid.

Baths were abandoned and doses of Sprudel salt ordered occasionally to keep the bowels soluble.

A year has now passed since treatment was finished and Mrs. S. continues in good health.

Case 3. A. Y., man, aged forty-five, resident of Newport,

R. I. Five years ago, contracted acute rheumatism from exposure, which finally became chronic from poor care and constant, necessary, hard work in a damp locality. Treatment was commenced a year ago, at which time the entire system was charged with uric acid. There was copious brick dust urinary deposits, concretions in finger joints, firm contractions of forearm muscles, "main-ewgriffe" and great weakness. In short, a more unpromising subject would be hard to find.

It was an evident fact that nothing could be done for the diseased condition until the man was placed in a more favorable condition, and by persistent effort he was secured admission into one of the charitable institutions of the State, where he was well fed and comfortably housed, with an opportunity for all needful medical care later.

When he began to grow strong, and show some signs of desire to live, he was placed on the Carlsbad water cure, steam bath plan. For the first two weeks no water was given internally, and but one bath a day, with plentiful nourishment.

On the fifteenth day he was given four half pint tumblers of Carlsbad water at a temperature of 100° F., which being well borne, were increased to six daily within another week, and the baths doubled.

Improvement followed the fourth week, and the water cure was suspended for a month, to allow nourishment to be pushed, when it was again resumed and continued for a month. With these intervals for rest, the Carlsbad treatment was followed a year, with the comfortable result of restoring Mr. Y. to such comparative health that he is able to do light work and be of some productive value in the world.

Case 4. Mr. G., aged fifty, independent gentleman, residence Providence, R. I., consulted me in 1889 for gout of the sub-acute form.

Digestion had become seriously impaired, and pain of a nagging kind was scarcely ever absent from the legs and feet.

Fever ran quite high every night, and the urine was loaded with particles of gravel, none large enough to cause serious interference with micturation, but all, when placed under the microscope, of sufficient size and sharpness of angles to account for the irritation that was present in the urethra.

Mr. G. came to me to be treated for nervous exhaustion, but upon making the necessary examination I decided that the exhaustion was dependent entirely upon his gouty condition, and placed him upon the treatment by Carlsbad water and Sprudel bath. He was forbidden the use of wines and stimulating food, and given a sufficient quantity of Sprudel salts each morning to insure a free evacuation of the bowels. No tonics were ordered, and he was directed to abstain from all exercise. The diet list was brought down to the simplest possible food, and his hours of eating changed from breakfast at eleven and dinner at eight, to those usually customary in America. After a week of this functional rest he was directed to take three tumblers of the water, slowly, at six o'clock, eight o'clock, and ten o'clock in the morning; at eleven a vapor bath was administered, and two hours rest followed, and a light breakfast of farinaceous food. A carriage ride of an hour or two occupied the greater part of the afternoon, and after a light nourishing dinner at six o'clock he was ordered to retire to bed and retain a recumbent posture until five the next morning; at that hour massage was administered thoroughly, followed by a tumbler of hot milk. This regime was continued for five weeks, at the expiration of which time a careful examination was made of blood and urine without finding any trace whatever of uric acid. His gout had disappeared, and the joints of the toes, which had commenced to enlarge, became flexible, and began to diminish in size. He was then permitted to add to his diet, lean meat of any kind and claret wine. The one tonic employed was general faradization a half hour daily. His condition promptly improved, and in one year after the cessation of all treatment, Mr. G. was in the most vigorous possible health, and made a long visit to Europe, whence he returned, I regret to say, in about the same condition as when he first consulted me, with the exception that there were then actual attacks of gravel, and I found that he had passed two or three well-formed uric acid calculi. I placed him at once upon the same treatment as before, with the same gratifying result, and am under the impression that a repetition will be necessary as often as cure is attained. The case, however, is one which shows in the strongest possible light, the remarkable and rapid gain in these conditions from the use of Carlsbad water and Sprudel salt.

Case 5. Mrs. E., aged thirty-seven, American, came to me in October of last year for a nervous trouble, supposed to be reflex from irritated ovaries, and probably salpingitis. She was anæmic, nervous to a very high degree, with capricious appetite, and with all of the secretions in an abnormal condition.

She had used opiates to a considerable degree, and was fast becoming addicted to its use.

A careful examination failed to reveal any great amount of trouble of the ovaries or tubes, and what was present was diagnosed as being of a reflex character, as well as all of her abdominal pains. A careful examination of the water showed a remarkable excess of uric acid, and was loaded down with brick dust deposit. A carefully selected diet, general faradization every day, taking away of all opiates, plenty of regular exercise, together with three goblets of Carlsbad water every day, supplemented with a dose of Sprudel salts every morning to keep the bowels soluble and regular, completed a perfect cure in three months time. She is now wholly well and hearty, and seven months pregnant.

It would not be difficult to multiply cases, but these five seem to me so fairly illustrative of the use and worth of these waters when far from the place where bottled and so good a showing of my methods of using them in vapor, that I submit them without further remark.—*N. E. Medical Monthly.*

## Figures and Tables

**Figure f1:**